# Addressing loneliness in emerging adults in primary care: a pilot feasibility study

**DOI:** 10.3389/fpsyt.2025.1470725

**Published:** 2025-05-15

**Authors:** Kris Pui Kwan Ma, Brennan Keiser, Melissa Garcia, Chialing Hsu, Karina Cortez, Ashley Johnson, Ajla Pleho, Mary C. Curran, Kelly Schloredt, Kwun C. G. Chan, Kari A. Stephens, Sebastian T. Tong

**Affiliations:** ^1^ Department of Family Medicine, University of Washington, Seattle, WA, United States; ^2^ Department of Psychology, University of Washington, Seattle, WA, United States; ^3^ Department of Rehabilitation Medicine, University of Washington, Seattle, WA, United States; ^4^ Department of Biostatistics, University of Washington, Seattle, WA, United States

**Keywords:** loneliness, young adults, emerging adults, primary care, cognitive behavioral therapy, social prescribing

## Abstract

**Introduction:**

Loneliness among emerging adults is common and is associated with poor physical and mental health. Most loneliness interventions have not been adapted nor tested in primary care that can broadly reach this population. This study aims to pilot test the feasibility, acceptability, and preliminary impact of two adapted interventions –cognitive behavioral therapy (CBT) and social prescribing (SP) – on reducing loneliness in emerging adults in primary care.

**Methods:**

Participants aged 18-25, who were seen in primary care and met the cut-off score on the UCLA-3 loneliness, were assigned to either CBT (N=6) or SP (N=9). Both group interventions were delivered virtually for five weeks. Outcomes included the 20-item UCLA loneliness scale, PHQ-9 depression, and GAD-7 anxiety. Ten qualitative interviews were conducted to understand participants’ experience of the interventions and effects on their loneliness.

**Results:**

Of 15 participants (11 women, mean age = 22), 14 of them completed either intervention. Results from paired T-tests showed pre-post reductions in loneliness, depression, and anxiety for both CBT and SP interventions, though they were statistically non-significant. Four themes described participants’ i) experience of loneliness, ii) changes in self and behavior, iii) barriers and facilitators to participation, and iv) suggestions for intervention adaptations.

**Discussions:**

The results suggest that it may be feasible to treat loneliness in emerging adults in primary care with adapted interventions like CBT and SP. Further research with larger sample sizes and pragmatic, randomized controlled trial designs are needed to test the effectiveness of these interventions in primary care settings.

## 1 Introduction

Loneliness – defined as a subjective feeling that one’s social relationships are deficient in some meaningful way (1) – has become increasingly prevalent in the U.S (2, 3). Emerging adults reported higher prevalence rates of loneliness than other age groups (4–7). Emerging adulthood is a critical life stage where individuals may be more prone to the risks of loneliness, due to life transitions and new developmental tasks that involve changes to their social networks (8, 9). Unaddressed loneliness in emerging adults is associated with poorer physical and mental health, including hypertension, anxiety and depression (10), substance use (11), poor sleep (12), and long-term mental illness (13).

Primary care is the first point of entry within the healthcare system that may be well positioned to detect and treat loneliness (14). Prior research suggests that patients with loneliness are often seen in primary care and that their visits to physician offices increase with their loneliness (15, 16). With appropriate interventions, primary care providers can play a key role in intervening early to address loneliness in emerging adults and potentially prevent the development of illnesses later in life.

Meta-analyses have reported that cognitive behavioral therapy (CBT) are effective in reducing loneliness with the preponderance of studies conducted in older adults (17–20), and its acceptability and treatment adherence vary greatly across different settings (17, 21). Social prescribing (SP) interventions have been successfully implemented in outpatient settings in European nations to help individuals strengthen their social networks and engage in purposeful activities, yet it is unknown which components of SP are most effective (22). In addition, SP has not been broadly studied with emerging adults and in the United States (23, 24). In young adults, one meta-analysis reported that interventions to date have focused on subgroups of youth who are at risk with loneliness as a secondary aim (25). In fact, to the best of our knowledge, neither CBT or SP loneliness interventions for emerging adults have been tested or widely implemented in primary care (21), where a broader population can be reached.

Our study aimed to examine the feasibility, acceptability and preliminary impact of CBT and SP interventions that were adapted for primary care settings to reduce loneliness in emerging adults.

## 2 Methods

### 2.1 Study design

The study used a pre-post testing design to assess differences in loneliness and other mental health outcomes in participants before and after interventions. Participants were assigned to the cognitive behavioral therapy (CBT) group or the social prescribing (SP) group. The University of Washington Institutional Review Board approved the study (STUDY00018180).

### 2.2 Recruitment and sample

Participants were between ages 18-25, spoke English, met the cutoff score for loneliness (scored ≥ 6 on the UCLA 3-item Loneliness scale) (26), had at least one primary care visit between July 2022 - October 2023 in a health system in Washington state, and were willing to participate in virtual group-based interventions. The health system is an academically affiliated urban health system with 16 primary care practices. Individuals who were receiving CBT treatments at the time of recruitment were excluded. As this was a pilot feasibility trial, the study was not powered to detect significant differences between two conditions.

### 2.3 Procedures

We used electronic health records (EHR) data to identify emerging adult patients in our primary care health system. We contacted the patients via text messaging and invited them to complete a REDCap survey to assess their eligibility. The survey included the UCLA-3 item loneliness scale (27) and participants’ demographics (i.e., age, gender, sex at birth, race and ethnicity, sexual orientation, relationship status, and living arrangements). Patients received $15 incentives for survey completion. We texted those who were eligible a link for electronic consent to join the study. Patients who provided consent and completed the baseline assessment were then assigned to either CBT or SP interventions using a sequential assignment method, where patients were assigned to groups based on the availability of open slots in each group following a predetermined sequence, to ensure they were balanced and even groups. Two CBT groups and two SP groups took place between fall 2023 – winter 2024.

### 2.4 Interventions

#### 2.4.1 Cognitive behavioral therapy

CBT is a psychological intervention that has shown efficacy and effectiveness in reducing loneliness and social isolation (17, 28, 29). CBT targets unhelpful thoughts and behaviors that are linked to loneliness and provides skills to improve social participation and relationships. Our brief 5-session CBT intervention is based on prior CBT protocols on loneliness and youth depression (19, 30) and was adapted to reach groups of emerging adults in primary care, after discussions with CBT experts and adolescent/young adult psychologists to focus on the core CBT components that target loneliness in this population. Core components of the intervention are summarized in **Table 1**. The CBT intervention was delivered in groups of 2–5 by two trained master-level psychotherapists over a videoconferencing platform weekly for 5 weeks; each session lasted for about 75 minutes.

**Table 1:** Summary of CBT and SP intervention sessions.

Sessions	Cognitive Behavioral Therapy (CBT)
1	*Psychoeducation:* Identify behaviors that are linked to loneliness
2	*Value identification and goal setting*: Develop new behaviors that facilitate meaningful social connections
3	*Behavioral activation and positive coping*: Strategies to overcome avoidance, passivity, and anxiety
4	*Cognitive reframing*: Work with thoughts that are linked to loneliness
5	*Relapse prevention*: Develop plans to maintain improvements and new behaviors
Sessions	Social Prescribing (SP)
1	Social world mapping: Starting a conversation about the person’s connections and understanding their social world
2	Creating connection plans: Setting goals to connect, reconnect, explore, and join in
3	Measuring success, social connection framework, and relationship with oneself
4	Overcoming barriers to social connections
5	Sustaining the plan: how to move forward

#### 2.4.2 Social prescribing

Social prescribing is an intervention that aims to reduce loneliness and associated symptoms of anxiety and depression by enhancing social networks. This intervention is based on evidence-based social prescribing practices that occur in primary care in the United Kingdom (31) and was adapted to reach groups of emerging adults after discussions with SP experts to identify core elements of social prescribing and conversations with psychologists with expertise in young adults to target the intervention to emerging adults. Core components of the intervention are summarized in **Table 1**. The SP intervention was delivered in groups of 4–5 by two facilitators over a videoconferencing platform weekly for 5 weeks; each session lasted about 60 minutes. Facilitators were not licensed psychotherapists but received training in delivering the SP intervention.

### 2.5 Assessments

Participants completed baseline and post-intervention surveys on REDCap. The baseline surveys were administered prior to assignment. The post-intervention surveys were administered within two weeks following the completion of the intervention. Participants received $25 for completing the baseline survey and $50 for completing the post-intervention survey. Both baseline and post-intervention surveys included the UCLA Loneliness-20, PHQ-9, and GAD-7.

### 2.6 Outcomes

The primary outcome was loneliness measured by the 20-item UCLA loneliness scale (version 3) (32). Using a 4-point rating scale (1= never; 4 = always), participants were asked to answer 20 questions on “how often they feel” about a positive or negative description of social interactions and perceptions. Scores range from 20-80, with higher scores indicating greater degrees of loneliness: 20-34 (low), 35-49 (moderate), 50-64 (moderately high), 65-80 (high) (33).

Secondary outcomes were depression and anxiety. Depressive symptoms were measured using the Patient Health Questionnaire-9 (PHQ-9) (34). Using a 4-point rating scale (0= not at all; 3 = nearly every day), participants were asked to measure symptoms of major depressive disorder over a 2-week period. Ratings are summed up and scores range are: 0-4 (minimal), 5-9 (mild), 10-14 (moderate), 15-19 (moderately severe), and 20-27 (severe). Anxiety symptoms were measured using the Generalized Anxiety Disorder 7-item Questionnaire (GAD-7) (35). Participants reported the presence and severity of anxiety symptoms over a 2-week period on a 4-point scale (0= not at all; 3 = nearly every day). Ratings were summed up and scores range are: 0-4 (minimal), 5-9 (mild), 10-14 (moderate), and 15-21 (severe).

To assess treatment adherence and participant engagement, both CBT and SP group facilitators completed a REDCap questionnaire for each participant after each session that included three questions: i) Did the participant attend the full treatment session? [Yes/no], ii) Overall, how much effort did the participant exert during group activities (e.g., in-group exercise, sharing, etc.)? and iii) How much was the participant engaged in the discussion and review of take-home practice? Group facilitators provided their subjective ratings on the latter two questions using a 5-point scale (1= none; 5= very often/very high effort). Ratings were summed up across all sessions for each participant and the total score ranged from 5 – 25, the higher the score, the greater the perceived level of participant’s engagement.

### 2.7 Qualitative interviews

All participants were offered post-intervention interviews and 10 agreed to participate. These participants had varying levels of participation in the interventions. The individual interviews were conducted by our research staff using a semi-structured interview guide that asks about the experience of loneliness, barriers and facilitators to study activities, recommended adaptations for future intervention design, and behavioral changes after study participation. Each interview lasted about 30–45 minutes and participants received $50 after completing the interviews. The interviews were audio recorded, de-identified, and transcribed.

### 2.8 Analyses

Our final sample (N = 15) included participants who completed baseline survey, postintervention survey, and attended at least one session of the CBT or SP intervention. Descriptive statistics were used to analyze and aggregate data on participant demographics and their treatment adherence. Paired T-tests were conducted separately for CBT and SP groups to examine whether there were significant pre- and post-intervention differences in outcomes. Group comparisons (CBT vs. SP) were not conducted as it was beyond the scope of this pilot study.

For qualitative interview data, we used Dedoose-9.0.46 (36) for data management and content analysis (37). The analysists (AJ and KC) first familiarized themselves with the data by reading each of the transcripts. A codebook was developed based on the semi-structured interview guide and the transcripts. Initially, two transcripts were coded by AJ and KC to verify accurate application of the codes. Then, the remaining transcripts were coded independently, and the team met regularly to discuss any inconsistencies within the code application. Once consensus was reached, emerging themes supported with quotes were used to summarize the findings.

## 3 Results

### 3.1 Enrollment, attrition, and participant characteristics

**Figure 1:**
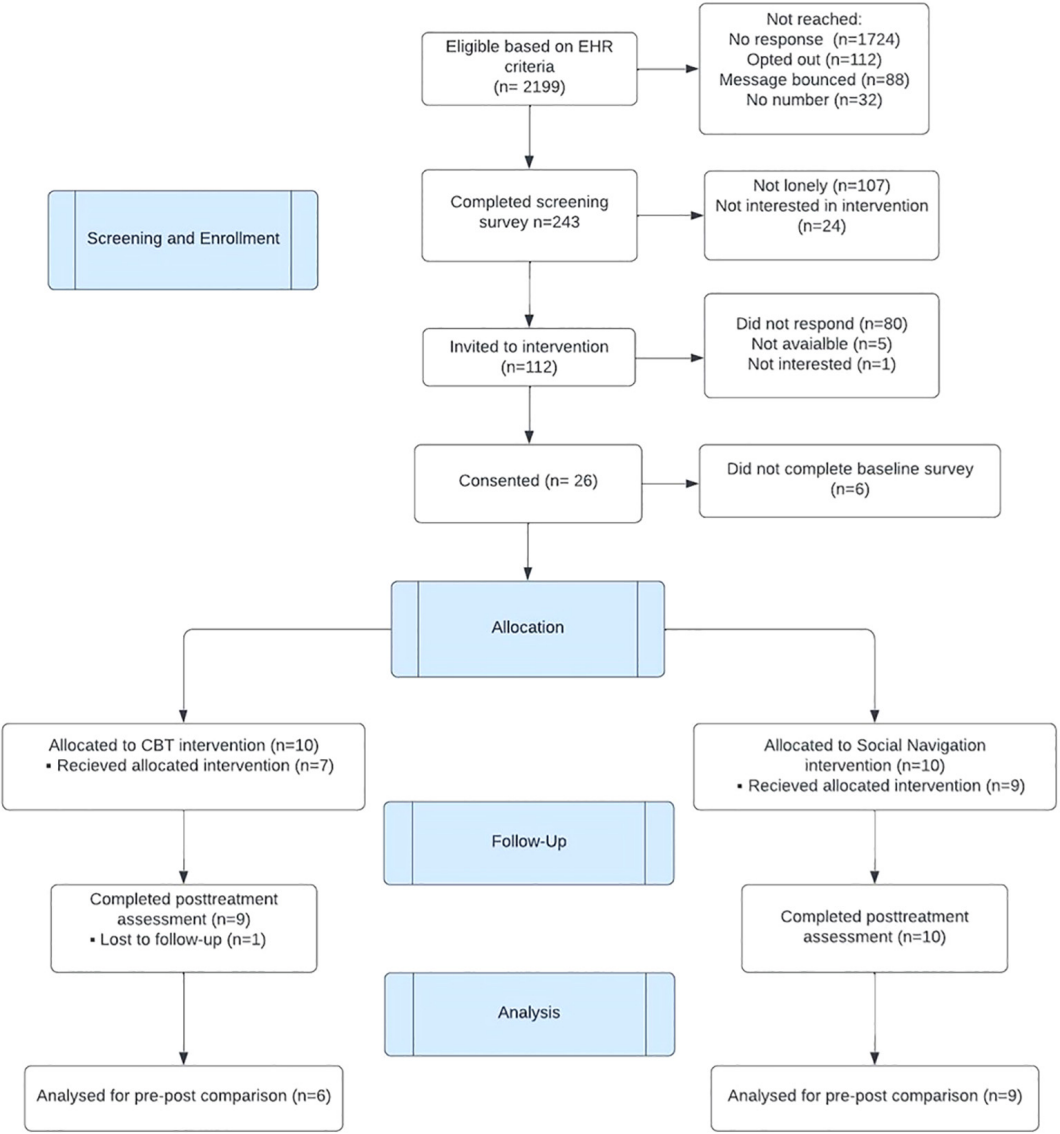
Flow diagram of participants through the study.

**Figure 1** depicts the flow diagram of participants through the study. Of 2,199 patients identified from the EHR, 243 (11%) of them completed the screening survey to assess eligibility. Of those who completed the screening, 112 (46%) were found eligible and expressed interest in participating in the study. The majority were excluded due to not meeting the cutoff score on UCLA-3 loneliness scale. Twenty-six (23%) eligible patients consented to enroll in the study; 20 of them completed the baseline assessment and were assigned to CBT (n = 10) and SP (n = 10) groups.

Overall attrition rate was at 25% (n = 5); four participants who were assigned did not start their assigned interventions (3 CBT, 1 SP) and one CBT participant did not complete the post-intervention survey. No significant demographic differences were found between those who dropped out vs. those who received the interventions. The five participants were excluded from the final analyses.

**Table 2** summarized the demographic characteristics of total participants that were included in the final pre-post analysis (N = 15). The average age was 22 (*SD* = 2.4). Most were female (87%) and identified as women (73%). Participants were diverse in their race, ethnicities, and sexual orientations (see **Table 2**).

**Table 2:** Demographics of participants (N = 15).

Characteristics	CBT (N=6)	SP (N=9)	Total (N = 15)
	mean (SD)/N (%)	mean (SD)/N (%)	mean (SD)/N (%)
Age	21.8 (2.1)	22.3 (2.6)	22.1 (2.4)
Race/Ethnicity			
American Indian/Alaskan Native	0 (0%)	0 (0%)	0 (0%)
Asian	1 (16.7%)	2 (22.2%)	3 (20%)
Black/African American	1 (16.7%)	0 (0%)	1 (6.7%)
Middle East and North African	0 (0%)	1 (11.1%)	1 (6.7%)
Native Hawaiian/Other Pacific Islander	0 (0%)	0 (0%)	0 (0%)
White	2 (33.3%)	4 (44.4%)	6 (40%)
Multiracial	1 (16.7%)	1 (11.1%)	2 (13.3%)
Other	0 (0%)	1 (11.1%)	1 (6.7%)
Hispanic or Latino	1 (16.7%)	0 (0%)	1 (6.7%)
Sex			
Female	5 (83.3%)	8 (89%)	13 (86.7%)
Male	1 (16.7%)	1 (11%)	2 (13.3%)
No Answer	0 (0%)	0 (0%)	1 (5.3%)
Gender Identity			
Man	1 (16.7%)	2 (22.2%)	3 (20%)
Woman	4 (66.7%)	7 (77.8%)	11 (73.3%)
Non-binary	1 (16.7%)	0 (0%)	1 (6.7%)
Sexual Orientation			
Asexual	1 (16.7%)	1 (11.1%)	2 (13.3%)
Bisexual, pansexual or queer	2 (33.3%)	4 (44.4%)	6 (40%)
Gay or lesbian	0 (0%)	1 (11.1%)	1 (6.7%)
Straight or heterosexual	3 (50%)	2 (22.2%)	5 (33.3%)
Not sure	0 (0%)	1 (11.1%)	1 (6.7%)
Other	0 (0%)	0 (0%)	0 (0%)
Relationship Status			
In a relationship	1 (16.7%)	1 (11.1%)	2 (13.3%)
Single	5 (83.3%)	8 (88.9%)	13 (86.7%)
Married	0 (0%)	0 (0%)	0 (0%)
Live Alone			
Yes	0 (0%)	2 (22.2%)	2 (13.3%)
No	6 (100%)	7 (77.8%)	13 (86.7%)
Live with Partner			
Yes	2 (33.3%)	1 (11.1%)	3 (20%)
No	4 (66.7%)	8 (88.9%)	12 (80%)
Live with Pet			
Yes	3 (50%)	4 (44.4%)	7 (46.7%)
No	3 (50%)	5 (55.6%)	8 (53.3%)
Live with Family			
Yes	4 (66.7%)	4 (44.4%)	8 (53.3%)
No	2 (33.3%)	5 (55.6%)	7 (46.7%)
Live with Roommate			
Yes	1 (16.7%)	2 (22.2%)	3 (20%)
No	5 (83.3%)	7 (77.8%)	12 (80%)

### 3.2 Treatment adherence and participant engagement

Of the 15 participants, 14 (93%) completed at least three out of five sessions in either intervention. Six (40%) participants completed all five treatment sessions: three each from the CBT and SP groups. The group facilitator-rated levels of participant effort and engagement were high in both groups. CBT participants were rated with mean scores of 19.7 and 19.3 (out of 25) for engaging in group activities and in take-home practice, while the SP participants were rated with mean scores of 16.6 and 17 respectively.

### 3.3 Pre vs. post differences

Results from paired t-tests showed a decrease in participants’ scores on loneliness, depression, and anxiety symptoms at post-intervention as compared to baseline for both interventions (see **Table 3**). The pre-post reductions in all outcomes were statistically non-significant, but the large reductions in UCLA-20 loneliness (*d* = -1.09) and in GAD-7 (*d* = -1.08) in CBT group suggested a potentially substantial magnitude of change and might be considered as clinically meaningful differences (28, 38, 39) (see **Table 3**).

**Table 3:** Paired T-tests for pre-post differences in CBT and SP interventions.

Timepoints	CBT (N=6)			SP (N=9)		
	Mean (*SD*)	*P-value*	*Cohen’s d*	Mean (*SD*)	*P-value*	*Cohen’s d*
UCLA-20 Loneliness						
pre	38.0 (14.4)			27.6 (8.4)		
post	24.8 (14.8)			24.0 (8.5)		
Difference	13.2	0.15	-1.09	3.6	0.39	-0.35
PHQ-9 Depression						
pre	12.7 (5.2)			12.4 (6.4)		
post	8.2 (3.3)			8.7 (3.5)		
difference	4.5	0.11	-0.10	3.7	0.15	-0.60
GAD-7 Anxiety						
pre	14.5 (3.8)			8.2 (5.2)		
post	10.3 (4.5)			6.6 (2.7)		
difference	4.2	0.11	-1.08	1.6	0.41	-0.32

### 3.4 Qualitative themes

Ten interviews were completed (4 CBT and 6 SP). Four themes emerged including the experience of loneliness, participant reported changes in self and behavior, barriers/facilitators to participation, and adaptations to the intervention design.

#### 3.4.1 Experience of loneliness

Participants described feelings of loneliness tied to emerging adult transitions such as moving cities, starting a new educational program, or adapting to a new living situation. Participants expressed loneliness as lacking meaningful connection to others or something external to themselves. The participants also illustrated how dynamic their experiences of loneliness as being influenced by individual mental health, partnership status, and availability of friends.

*“But at the end of the night, everyone goes back to their dorm, and they’re just like alone … I went from going to school with people and seeing people every single day to seeing people, but like I don’t really know them. And we’re not like friends and having like a lot of alone time in my dorm just by myself.” (CBT participant)*

#### 3.4.2 Participant reported changes in self and behavior

Participants in both CBT and SP groups reported that hearing the experience of others normalizes their own experience of loneliness. They described a shift in their perspective-taking as they discovered commonality in their feelings with other group members and gained insight into their own internal self-dialogue of being critical. They reported developing self-compassion and an increased understanding of self, which empowered them to recognize their feelings of loneliness and act on things that they can control. Participants in both groups reported increased confidence in social interaction and a newfound sense of control to engage in activities that improve their loneliness.

“*I have more self-confidence with engaging with people and interacting. I also feel less frustrated with myself. This helps shift my focus to what I can do instead of what I can’t do. And so now, instead of being the person who’s consistently [saying] I can’t make it to this event. (CBT participant)*

CBT participants reported gaining skills of exploring emotions, understanding their origins and identifying strategies for managing them. They learned to explore personal motivation or triggers for isolating oneself and gain insights into the underlying factors. They acknowledged the significance of addressing negative self-talk and transforming it to positive self-talk as a way to combat loneliness. They also employed relaxation techniques when reaching out to others, engaging in social activities, and managing their reactions to unpleasant social situations (e.g., someone’s absence or inability to reciprocate).

“*I do have that kind of mental memo now where I’ll yeah be like having a conversation with somebody and notice that I’m feeling kind of differently and be like, stop and breathe- internal-you know I have that now … And it has been beneficial also relaxation technique.” (CBT participant)*

SP participants found that setting goals is important for making change and that tracking personal success and having accountability supports that change. They discussed the importance of not only focusing on improving social skills but also actively engaging in social situations and practicing them in real life scenarios. Participants in this group also gained skills in recognizing opportunities to strengthen social connection through mapping out social network and engaging in activities related to hobbies which improved social experiences.

*“I would get that a lot of the ideas for how to connect with people is not going to be shocking, or like revolutionary, but it’s more about putting the work in and actually holding yourself to that standard, and then tracking the progress you’ve made, wow, this is like a new concept I haven’t really thought about before.”* (SP participant)

#### 3.4.3 Barriers and facilitators to participation

Overall, few barriers were reported by participants. There was mixed feedback regarding the format (virtual vs. in-person) for the sessions. The CBT group found the virtual format made attendance more accessible, while the SP group preferred a hybrid option. For instance, a SP participant found the virtual format to be difficult due to living with roommates.

“*Oh, I guess one barrier was just with my roommates … I was like trying not to be, you know too much in their* sp*aces, because since it was in the evening and that was kind of maybe the main one, just being in this* sp*ace here and I live with my partner and I did not want to take up the room from her and stuff.” (SP participant)*

*“I personally like their virtual option, because I don’t drive so I wouldn’t be able to make it if it was like an in-person thing. And I feel like talking about emotions in person in a group would just be kinda awkward and take a while to break the ice with strangers.” (CBT participant)*

Facilitators to both interventions included compassionate and engaging group facilitators and applicable session materials. Both groups expressed that the facilitators were welcoming of personal experiences, created a safe environment that fostered an inclusive atmosphere for discussion, and worked to maintain conversation momentum and meaningful participant interactions. Participants in both CBT and SP groups found the topics, activities and materials applicable and helpful.

“I just thought they were super like warm and welcoming and nonjudgmental and did a good job of engaging with us and responding to what we had to say and affirming our thoughts and feelings.” (SP participant)

#### 3.4.4 Adaptations to intervention design

Both groups thought that there could be more time to work with the materials in the session and ask questions. The resources provided in the intervention were helpful, actionable, and provoked reflection. They expressed interest in extended sessions to help maintain the flow of group discussion and to continue enjoy participating in the group sessions. They also expressed a preference for starting with a larger cohort in hopes of accounting for potential drops in attendance and absences. The CBT group expressed that the worksheets were burdensome and suggested using them more as a mental tool. The SP group mentioned that the online communication tool (Discord) was not utilized to its full potential.

“*I think the handouts are a good way of kind of thinking about the flow. If you need some reference to how to approach certain things or how to use some tools. The handouts are good. But I think, like some of us, that we work and stuff, we don’t really get the time to kind of sit and like fill them out.” (CBT participant)*

*“Um, I didn’t use it very much. I’m not someone who uses discord regularly. I kind of like forgot to be checking it regularly.” (SP participant)*

## 4 Discussion

The results showed promising indicators of feasibility and acceptability of implementing the adapted CBT and SP loneliness interventions in primary care, as demonstrated by the high percentage of emerging adult patients that were screened for loneliness and expressed interests in the interventions (46%), the high intervention completion rates for those who joined (93%), and the relatively low attrition rate (25%) comparing to prior studies (17, 21). However, enrollment challenges were seen in the low proportion of eligible patients (23%) who responded to our invitation and joined the study. Our enrollment numbers were relatively low compared to other loneliness intervention studies in primary care (for older adults) (40) and in community settings (for younger adults) (18). One possible explanation could be our sole use of EHR-based recruitment strategy. EHR allows for a quick and wide reach of diverse patient population for identification and screening but may not offer researchers adequate bonding opportunities with potential participants, which has been considered as a key ingredient by emerging adults to engage in interventions (41). In future studies, we plan to use a multi-pronged approach to recruitment and track the recruitment numbers of different strategies (EHR-based vs. clinician referrals vs. community outreach vs. combined), to compare their effectiveness and acceptability in recruiting emerging adult patients.

Participants found the topics, activities, and materials in CBT and SP interventions applicable and relevant. They noted that the compassionate group environment was an important facilitator for their participation and treatment gains. This sentiment echoed with prior qualitative research (41), which suggests that the group format may be a generally preferrable approach for emerging adults and that future research can investigate the role of group dynamics in loneliness interventions. On the other hand, participants had divergent views regarding the virtual delivery of interventions. They recognized the accessibility and convenience of remote and online interventions, but also desired the incomparable social fulfillment found in in-person interactions. Currently there is no conclusive evidence on which delivery mode is superior in terms of outcomes, yet concerns around physical location of intervention delivery, particularly in rural areas, have been raised in prior reports (40). These mixed findings necessitate the future exploration of the optimal delivery mode for primary care-based loneliness interventions that aim to reach and engage emerging adults.

Despite lack of significant pre-post differences detected in our study, we observed improvements in all outcomes across the intervention period for both groups, especially with potentially large reductions in loneliness and anxiety for CBT group and depression for SP group. Our preliminary findings seem consistent with prior literature that found large effect sizes in depression and anxiety for group CBT interventions among emerging adults and university students (38, 42). Evidence for CBT in loneliness reduction is less conclusive (43). While a meta-analysis reported a small to medium pooled effect size for CBT interventions in reducing loneliness across age groups (17), our study found a large loneliness reduction for the CBT group, which coincides with the findings of two recent studies outside of the U.S. that focused specifically on CBT for loneliness in young people in community and university settings (20, 38). Only a handful of studies have examined SP interventions for reducing depression and loneliness in emerging adults and university students and have shown promising results (43, 44). More research is needed to determine the evidence for SP effectiveness (45).

The qualitative reports highlighted participants’ perspectives on their changes in self-perceptions, behaviors, skills, and social networks, which all may be important mechanisms of change for reducing loneliness. Their nuanced and experiential perspectives also indicated the different strategies used in CBT (modify thinking patterns and behaviors) vs. SP (increase opportunities for social contact), as illustrated in Pearce et al. (2021)’s conceptual framework (46) of loneliness interventions in young people. Given emerging adults have heterogeneous experience of loneliness, examining which aspects and combinations of loneliness interventions may be most effective and feasible in primary care can help target the scope and types of loneliness that are being treated in primary care for this population.

### 4.1 Limitations and future research

Given the exploratory nature of this pilot feasibility study, the findings should be interpreted in the context of three study limitations. First, the study used a small convenience sample of emerging adults identified from the EHR data in a large urban health system; hence there could be sampling bias, and the results may not be generalizable. Future studies will include larger samples that are powered to detect within- and between-group differences in intervention outcomes. Second, a control group was not included and thus we cannot conclude participants’ reported changes in loneliness, depression and anxiety were due to the intervention and not to other causes. There were also potential allocation and temporal biases with the sequential assignment method, which could result in unequal group characteristics unrelated to the intervention. Our future study will use a randomized controlled trial design to examine the treatment effects of these interventions against the control group and will include longitudinal follow-ups as well as analyses controlling for covariates. Third, challenges with regular attendance and varying levels of engagement may have affected outcomes; this was not analyzed due to the small sample size. Despite the limitations, results from this pilot work are informative to refine the interventions and to prepare for a future RCT.

## 5 Conclusion

This pilot study adds valuable knowledge about the possibilities of alleviating loneliness in emerging adults in primary care by means of group-based interventions using CBT and SP. While more research is needed on establishing intervention effectiveness and optimizing recruitment and engagement strategies, the results indicated that both CBT and SP interventions may be feasible, acceptable, and may be associated with reductions in loneliness among emerging adults in primary care.

## Data Availability

The original contributions presented in the study are included in the article/Supplementary Material. Further inquiries can be directed to the corresponding author.
